# Supplementary material for: A Critical View of the Application of the APEX Software (Aqueous Photochemistry of Environmentally-Occurring Xenobiotics) to Predict Photoreaction Kinetics in Surface Freshwaters
Source: Molecules. 2019 Dec 18;25(1):9. doi: 10.3390/molecules25010009 (PMC7017383; doi:10.3390/molecules25010009)
Supplement: Supplementary file 1 [file molecules-25-00009-s001.zip › 3.2.4_gcc-4.4.0.zip!/3.2.4_gcc-4.4.0/3.2.4_gcc-4.4.0/doc/octave/html/interpreter/Variables.html]

Variables - Untitled


Next: Expressions,
Previous: Data Containers,
Up: Top


---

## 7 Variables

Variables let you give names to values and refer to them later. You have
already seen variables in many of the examples. The name of a variable
must be a sequence of letters, digits and underscores, but it may not begin
with a digit. Octave does not enforce a limit on the length of variable
names, but it is seldom useful to have variables with names longer than
about 30 characters. The following are all valid variable names

```
     x
     x15
     __foo_bar_baz__
     fucnrdthsucngtagdjb
```

However, names like `__foo_bar_baz__` that begin and end with two
underscores are understood to be reserved for internal use by Octave.
You should not use them in code you write, except to access Octave's
documented internal variables and built-in symbolic constants.

Case is significant in variable names. The symbols `a` and
`A` are distinct variables.

A variable name is a valid expression by itself. It represents the
variable's current value. Variables are given new values with
assignment operators and increment operators.
See Assignment Expressions.

There is one built-in variable with a special meaning. The `ans` variable
always contains the result of the last computation, where the output wasn't
assigned to any variable. The code `a = cos (pi)` will assign the value -1
to the variable `a`, but will not change the value of `ans`. However,
the code `cos (pi)` will set the value of `ans` to -1.

Variables in Octave do not have fixed types, so it is possible to first
store a numeric value in a variable and then to later use the same name
to hold a string value in the same program. Variables may not be used
before they have been given a value. Doing so results in an error.

— Automatic Variable: **ans**  
> The most recently computed result that was not
> explicitly assigned to a variable. For example, after the expression
>
> ```
>           3^2 + 4^2
> ```
>
> is evaluated, the value returned by `ans` is 25.

— Built-in Function: **isvarname** (name)  
> Return true if name is a valid variable name

— Function File: varname = **genvarname** (str)  
— Function File: varname = **genvarname** (str, exclusions)  
> Create unique variable(s) from str. If exclusions is
> given, then the variable(s) will be unique to each other and to
> exclusions (exclusions may be either a string or a cellstr).
>
> If str is a cellstr, then a unique variable is created for each
> cell in str.
>
> ```
>           x = 3.141;
>           genvarname ("x", who ())
>           => x1
> ```
>
> If wanted is a cell array, genvarname will make sure the returned
> strings are distinct:
>
> ```
>           genvarname ({"foo", "foo"})
>           =>
>           {
>             [1,1] = foo
>             [1,2] = foo1
>           }
> ```
>
> Note that the result is a char array/cell array of strings, not the
> variables themselves. To define a variable, `eval()` can be
> used. The following trivial example sets `x` to `42`.
>
> ```
>           name = genvarname ("x");
>           eval([name " = 42"]);
>           => x =  42
> ```
>
> Also, this can be useful for creating unique struct field names.
>
> ```
>           x = struct ();
>           for i = 1:3
>             x.(genvarname ("a", fieldnames (x))) = i;
>           endfor
>           =>
>           x =
>           {
>             a =  1
>             a1 =  2
>             a2 =  3
>           }
> ```
>
> Since variable names may only contain letters, digits and underscores,
> genvarname replaces any sequence of disallowed characters with
> an underscore. Also, variables may not begin with a digit; in this
> case an underscore is added before the variable name.
>
> Variable names beginning and ending with two underscores "\_\_" are valid but
> they are used internally by octave and should generally be avoided, therefore
> genvarname will not generate such names.
>
> genvarname will also make sure that returned names do not clash with
> keywords such as "for" and "if". A number will be appended if necessary.
> Note, however, that this does **not** include function names,
> such as "sin". Such names should be included in avoid if necessary.
>
>
>
> **See also:** isvarname, exist, tmpnam, eval.

— Function File: **namelengthmax** ()  
> Returns the matlab compatible maximum variable name length. Octave is
> capable of storing strings up to
> `2 ^ 31 - 1`
> in length. However for matlab compatibility all variable, function
> and structure field names should be shorter than the length supplied by
> `namelengthmax`. In particular variables stored to a matlab file
> format will have their names truncated to this length.

- Global Variables- Persistent Variables- Status of Variables
